# The added value of satellite observations of methane forunderstanding the contemporary methane budget

**DOI:** 10.1098/rsta.2021.0106

**Published:** 2021-11-15

**Authors:** Paul I. Palmer, Liang Feng, Mark F. Lunt, Robert J. Parker, Hartmut Bösch, Xin Lan, Alba Lorente, Tobias Borsdorff

**Affiliations:** ^1^ School of GeoSciences, University of Edinburgh, Edinburgh, UK; ^2^ National Centre for Earth Observation, University of Edinburgh, Edinburgh, UK; ^3^ Department of Physics and Astronomy, University of Leicester, Leicester, UK; ^4^ National Centre for Earth Observation, University of Leicester, Leicester, UK; ^5^ NOAA Global Monitoring Laboratory, Boulder, CO, USA; ^6^ SRON Netherlands Institute for Space Research, Utrecht, The Netherlands

**Keywords:** atmospheric methane, satellite observations, inverse modelling, methane emissions, surface measurements

## Abstract

Surface observations have recorded large and incompletely understood changes to atmospheric methane (CH_4_) this century. However, their ability to reveal the responsible surface sources and sinks is limited by their geographical distribution, which is biased towards the northern midlatitudes. Data from Earth-orbiting satellites designed specifically to measure atmospheric CH_4_ have been available since 2009 with the launch of the Japanese Greenhouse gases Observing SATellite (GOSAT). We assess the added value of GOSAT to data collected by the US National Oceanic and Atmospheric Administration (NOAA), which have been the lynchpin for knowledge about atmospheric CH_4_ since the 1980s. To achieve that we use the GEOS-Chem atmospheric chemistry transport model and an inverse method to infer *a posteriori* flux estimates from the NOAA and GOSAT data using common *a priori* emission inventories. We find the main benefit of GOSAT data is from its additional coverage over the tropics where we report large increases since the 2014/2016 El Niño, driven by biomass burning, biogenic emissions and energy production. We use data from the European TROPOspheric Monitoring Instrument to show how better spatial coverage and resolution measurements allow us to quantify previously unattainable diffuse sources of CH_4_, thereby opening up a new research frontier.

This article is part of a discussion meeting issue ‘Rising methane: is warming feeding warming? (part 1)’.

## 1. Introduction

Atmospheric methane (CH_4_) absorbs and emits radiation at infrared wavelengths and therefore plays a role in determining Earth’s radiative balance. It has a higher global warming potential than CO_2_; after carbon monoxide it is the principal sink of the hydroxyl radical (OH), which is the major oxidant in the global troposphere, and contributes to the production of tropospheric ozone, another important greenhouse gas. Consequently, it is an ideal target for rapid reductions to make substantive progress towards meeting the aims of the Paris Agreement [1,2]. For all of these reasons, it is a science priority to address our inability to attribute definitively recent and large changes in the global mass of atmospheric CH_4_ since the turn of the century [1,3,4]. In this study, we compare what we understand about recent changes (2010–2019) in global and regional CH_4_ emissions from ground-based data and from satellite column retrievals of CH_4_ at short-wave infrared (SWIR) wavelengths.

Observed changes in atmospheric CH_4_ are determined by surface emissions and by surface and atmospheric sinks [5]. The largest natural source is emissions from wetlands, with smaller natural emissions from freshwaters, onshore and offshore geological sources, wild animals, termites, permafrost soils, and open and coastal ocean. Anthropogenic emissions are dominated by agriculture, including enteric fermentation from ruminants, manure management and rice cultivation, and by waste management that includes the microbial decomposition of organic material in landfills. Emissions from fossil fuels are approximately half to two-thirds of those from agriculture and waste [5] and include coal mining, the oil and gas industry, and transport. Combustion of biomass and biofuel is also a significant source of CH_4_. The dominant loss process for CH_4_ is oxidation by tropospheric OH, with small losses from stratospheric loss, reaction with chlorine, and uptake from soils. The resulting steady-state atmospheric lifetime of CH_4_ is ≃9 years [6]. The perturbation lifetime of CH_4_, which accounts for atmospheric chemistry relaxation times and is more relevant for climate impacts of emission reductions, is approximately 12 years [7].

After decades of steady growth in the twentieth century, the atmospheric growth of atmospheric CH_4_ reduced to approximately zero from 2000 to 2006 [4], a consequence of the production and loss processes being in quasi-equilibrium. Atmospheric growth has since returned to values observed in the second half of the twentieth century [1,3] and more recently has increased at a faster rate. There is extensive debate in the literature about which sources are responsible for these recent observed global-scale changes [8–14], with some studies emphasizing that variations in the OH could also be responsible but this appears to be unlikely given the behaviour of other trace gases that are oxidized by OH [15,16]. A more likely scenario is that some combination of emission and loss variations are responsible for observed atmospheric variations in CH_4_.

**Figure 1.**
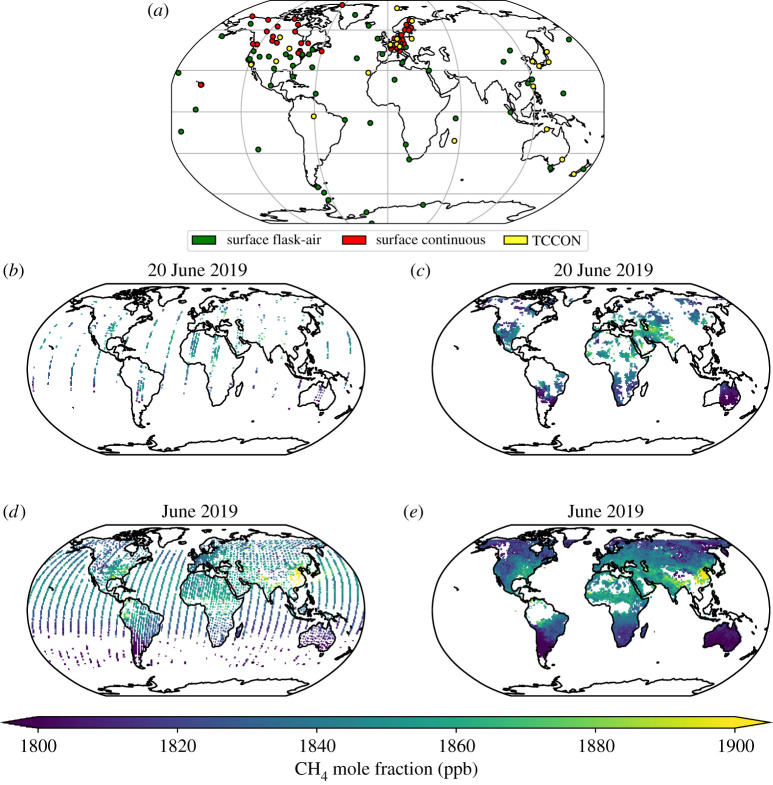
Geographical locations of data collected by (*a*) ground-based measurements operated by NOAA, daily distributions of clear-sky methane columns observed by (*b*) GOSAT and (*c*) TROPOMI satellite instruments on 20 June 2019, and the corresponding monthly distributions for (*d*) GOSAT and (*e*) TROPOMI for June 2019. Ground-based measurements include flask measurements (green dots) and *in situ* continuous analyser measurements (red dots) of CH_4_ operated by NOAA, a subset of which we use to determine *a posteriori* flux estimates, and the methane columns measured the Total Carbon Column Observing Network (TCCON, [17]). (Online version in colour.)

Calibrated atmospheric CH_4_ surface measurements have been collected across the globe (figure 1*a*) by a variety of groups, the most extensive network of which has been coordinated by the US National Oceanic and Atmospheric Administration (NOAA) since the 1980s. The original purpose of these measurements was to observe large-scale changes driven by natural and anthropogenic emissions, although the network has grown with time and a growing body of work (including this study) have used these data to infer continental-scale emission estimates. The preponderance of these measurement sites, taking advantage of sites established to collect CO_2_ measurements, are over North America and Europe and that has implications for understanding sub-continental changes in CH_4_ emissions. Colocated measurements of CH_4_ isotopologues provide additional information with which to improve source attribution (e.g., [3,12,18–21]). In particular, progressively lighter measurements of $\delta^{13}C_{{\mathrm{CH}}_{4}}$ suggest that recent changes in atmospheric CH_4_ are due to increased biological activity, e.g. [3,12,22].

Data from the European SCIAMACHY (SCanning Imaging Absorption spectroMeter for Atmospheric CartograpHY) satellite instrument [23], launched in 2002, were the first space-borne measurements that were sensitive to changes in boundary layer CH_4_ [24]. Serious degradation of detector pixels from the end of 2005 compromised these data for quantifying regional CH_4_ fluxes [25], although they provided invaluable information about year-to-year variations in atmospheric CH_4_ [26]. The Japanese Greenhouse gases Observing SATellite (GOSAT) has collected data since it was launched in 2009 [27]. The main advantage of using satellite data is the global coverage they provide (figure 1*b*,*d*), although instruments are typically in a sun-synchronous orbit so they sample the atmosphere at one local time of the sunlit day. The SWIR wavelengths used to determine CH_4_ columns that are sensitive to the lower troposphere are also sensitive to clouds so columns are usually only retrieved in cloud-free scenes, and the columns are difficult to interpret without a model of atmospheric chemistry and transport. Ground-based upward looking spectrometers, e.g. the Total Carbon Column Observing Network (TCCON, figure 1), play an ongoing key role in ensuring the accuracy of the satellite data [17]. GOSAT data have significantly revised our understanding of regional CH_4_ budgets across the globe, e.g. [9,10,28–37]. These include studies focused over the tropics where we have little other data available to revise our *a priori* knowledge, e.g. [9,10,28,30,32,36,37], for which in some examples the inferred emissions can be linked to specific source types, e.g. [9,10,32,36,38].

There remain many outstanding science questions associated with CH_4_ emissions, some of which are emerging as we witness more frequent anomalous climate variations while others are associated with our ability to detect changes in atmospheric CH_4_ that correspond to national net zero pledges. The ability of satellite data to help address these science questions will progressively improve with the length and density of data records, as newer instruments with improved detector technology and better spatial resolution become available. Here we take advantage of the decadal record of CH_4_ column data from GOSAT to explore the value of these data over and above the information provided by the NOAA *in situ* network, described in §2, to understand CH_4_ emissions on global to subcontinental spatial scales. We achieve this by inferring CH_4_ emissions from these data using common *a priori* inventories, and a common atmospheric chemistry transport model and ensemble Kalman filter inverse method, which are all described in §2. In §3, we report *a priori* and *a posteriori* CH_4_ fluxes inferred from NOAA and GOSAT CH_4_ data on global and continental spatial scales, with a specific focus on tropical South America and the Indian subcontinent. We conclude this section by examining the potential of finer resolution CH_4_ data from the European TROPOspheric Monitoring Instrument (TROPOMI) by quantifying diffuse coal mining emissions of CH_4_ over Northern Queensland, Australia. We conclude the paper in §4.

## 2. Data and methods

### (a) *In situ* mole fraction CH_4_ data

We use biweekly CH_4_ values determined from measurements of discrete air samples collected in flasks and from continuous online analysers from across the NOAA Cooperative Global Air Sampling Network (figure 1)

We also use CO_2_ measurements as part of our novel analysis of GOSAT CH_4_ proxy data, as described below. We use (weekly) discrete flask air samples from 105 sites and (hourly) continuous observations from 52 sites that are part of the global atmospheric surface CO_2_ observations network. These are currently described by the Observation Package (ObsPack) data products: obspack_co2_1_GLOBALVIEWplus_v5.0_2019-08-12 and (for late 2019) obspack_co2_1_NRT_v5.1.1_2020-03-05, provided by the NOAA Global Monitoring Laboratory.

### (b) Satellite data

We use data from the GOSAT instrument for our comparative analysis with NOAA *in situ* data, and data from TROPOMI to show how finer spatially resolved data can be used to infer diffuse emissions of CH_4_ from coal mining. Methane columns for GOSAT and TROPOMI (table 1) take advantage of SWIR wavelengths that are sensitive to changes in CH_4_ in the lower troposphere but also sensitive to cloud coverage so that we use only cloud-free scenes.

**Table 1.** Satellite instruments that have contributed to our understanding of atmospheric CH_4_ and the corresponding regional distribution of emissions. LECT refers to the local equatorial crossing time and the repeat frequency refers to the approximate time between successive measurements over a particular region, subject to clear-sky criteria.

	data	wavelength	orbit, LECT,	ground footprint
instrument	availability	coverage	repeat frequency	dimension
SWIR instruments				
SCIAMACHY (nadir)	2002–2012	SWIR	SS, 1000d, 3	$30\times 60\;{\mathrm{km}}^{2}$
GOSAT-1/TANSO	2009–present	SWIR/TIR	SS, 1300d, 3	10.5 km diameter
GOSAT-2/TANSO	2019–present	SWIR/TIR	SS, 1300d, 3	10.5 km diameter
TROPOMI	2018–present	SWIR	SS, 1330a, 1	$5.5\times 7\;{\mathrm{km}}^{2}$

### (c) Greenhouse gases Observing SATellite CH_4_ column measurements

GOSAT was launched in 2009 by the Japanese Space Agency (JAXA), in collaboration with the Japanese National Institute for Environmental Studies and the Ministry of Environment. The satellite is equipped with a high-resolution Fourier transform spectrometer (TANSO-FTS) that enables the measurement of concentrations of both CO_2_ and CH_4_. GOSAT is in a sun-synchronous orbit, with a local equator crossing time of 13.00. The instrument has a ground footprint with diameter of 10.5 km with a pixel spacing of approximately 250 km. GOSAT achieve approximate global coverage in three days.

We use GOSAT proxy column methane (XCH4) data from the University of Leicester (v. 9.0) [39,40], which has been validated against data from the TCCON network [34] and occasionally using regional aircraft data (e.g. [41]). The proxy XCH4 retrieval simultaneously retrieves CH_4_ and CO_2_ columns using absorption features around the wavelength of 1.6 μm. These columns are most sensitive to changes in CO_2_ and CH_4_ in the lower troposphere, where variations are sensitive to surface fluxes. Taking the ratio of these retrieved columns, ${\mathrm{CH}}_{4}/{\mathrm{CO}}_{2}$, effectively assumes CO_2_ is a proxy for modifications along the light path [25] and minimizes the influence of common factors that affect the retrieval of both gases, e.g. clouds and atmospheric scattering. Consequently, these ratios are less sensitive against scattering than a full-physics retrieval approach [42], resulting in higher data density over geographical regions where there is substantial aerosol loading, e.g. tropical dry seasons. Analyses have shown that these retrievals have a bias of 0.2%, with a single sounding precision of about 0.72% [34,40,43].

The conventional approach is then to scale the ratio with an independent estimate for the CO_2_ column, often from a model, to infer CH_4_ columns. This ratio is used to determine CH_4_ rather than CO_2_ because it is generally assumed that CO_2_ varies much less than CH_4_. But of course our knowledge of CO_2_ is incomplete (e.g. [44–46]), particularly over the tropics, so this last step introduces an unnecessary systematic error to the resulting CH_4_ columns [34]. We use an alternative approach, which we previously developed, to directly use the ${\mathrm{CH}}_{4}/{\mathrm{CO}}_{2}$ by taking advantage of sparsely distributed *in situ* that help anchor the GOSAT ratio data, allowing us to simultaneously infer CH_4_ and CO_2_ fluxes [28,29].

### (d) TROPOspheric Monitoring Instrument CH_4_ column measurements

The TROPOMI on board the Sentinel-5p satellite was launched in 2017. The satellite is in a sun-synchronous orbit with a local equator crossing time of 13:30. With a swath width of around 2600 km, it provides complete daily coverage of the globe at $5.5\times 7\;{\mathrm{km}}^{2}$ resolution, upgraded from $7\times 7\;{\mathrm{km}}^{2}$ in August 2019. The spectral range of TROPOMI precludes using the proxy retrieval approach so CH_4_ columns are determined by a full-physics approach that uses the CH_4_ absorption features around the wavelength of 2.3 μm [42,47,48], which takes into account aerosol and cloud scattering. We use the scientific CH_4_ data product [48]. These data include an *a posteriori* correction based on TROPOMI data to account for biases at high and low albedos, following [49]. These column data have been validated against TCCON and GOSAT data, with a mean bias (standard deviation) with TCCON of −3.4 (5.6) ppb, and values of −10.3 (16.8 ppb) compared to GOSAT [48].

### (e) GEOS-Chem global three-dimensional atmospheric chemistry transport model

For the experiments reported here, we use the GEOS-Chem atmospheric chemistry and transport model at a horizontal resolution of $4^{\circ }$ (latitude)$\;\times \;5^{\circ }$ (longitude), driven by the MERRA-2 meteorological re-analyses from the Global Modelling and Assimilation Office Global Circulation Model based at NASA Goddard Space Flight Center. This model is used to relate *a priori* emissions to four-dimensional atmospheric fields of CH_4_. We also describe *a priori* fluxes for CO_2_ that we need to infer simultaneously fluxes of CH_4_ and CO_2_.

Our *a priori* CO_2_ flux inventory includes: (1) monthly biomass burning emission (GFEDv4.1) [50]; (2) monthly fossil fuel emissions (ODIAC) [51]; (3) monthly climatological ocean fluxes [52]; and (4) 3 h terrestrial biosphere fluxes (CASA) [53]. Our CO_2_ model calculations follow closely a recent study [46]. Our *a priori* CH_4_ fluxes from nature include: (1) monthly WetCHARTS v1.0 wetland emissions, including rice paddies [54]; (2) monthly fire CH_4_ emissions are from GFEDv4.0; (3) termite emissions [55]. Emissions from geological macroseeps are based on [56] and [57]. For areal seepage, we use the sedimentary basins (microseepage) and potential geothermal seepage maps [57] with the emission factor described by [58]. For *a priori* anthropogenic emissions, we use the EDGAR v4.41 global emission inventory [59] that includes various sources related to human activities (e.g. oil and gas industry, coal mining, livestock and waste). We use monthly three-dimensional fields of the hydroxyl radical, consistent with observed values for the lifetime of methyl chloroform, from the GEOS-Chem HOx-NOx-Ox chemistry simulation [35,60] to describe the main loss of tropospheric CH_4_ [61] and the loss of CH_4_ in the stratosphere. Using fixed, archived field of OH allows us to linearly decompose total CH_4_ into contributions from individual sources and geographical regions. We also include a simple soil sink of CH_4_ [61].

### (f) Ensemble Kalman filter inverse method

We use an ensemble Kalman filter (EnKF) framework [28] to estimate simultaneously CO_2_ and CH_4_ fluxes from and satellite measurements of the atmospheric CO_2_ and CH_4_ from 2009 to 2019, inclusively. For these experiments, we report net CH_4_ emission estimates and do not attempt to distinguish emissions from individual sectors.

Our state vector includes monthly scaling factors for 486 regional pulse-like basis functions that describe CO_2_ and CH_4_ fluxes, including 476 land regions and 11 oceanic regions. We define our land sub-regions by dividing the 11 TransCom-3 [62] land regions into 42 nearly equal sub-regions, with the exception for temperate Eurasia that has been divided into 56 sub-regions due to its large landmass. We use the 11 oceanic regions defined by the TransCom-3 experiment.

We assume the *a posteriori* CH_4_ or CO_2_ flux estimate takes the form [28]:

2.1 $$f_{p}^{g}(x,t)=f_{0}^{g}(x,t)+\sum_{i}c_{i}^{g}BF_{i}^{g}(x,t),$$

where g denotes the atmospheric concentration of CH_4_ or CO_2_ and $f_{0}^{g}(x,t)$ and $f_{p}^{g}(x,t)$ describe their *a priori* and *a posteriori* flux inventories, respectively. The pulse-like basis functions $BF_{i}^{g}(x,t)$ represent the sum of different source sectors, which we use to represent their overall spatial pattern for each month over each sub-region. $c_{i}^{g}$ denotes the state vector that comprises of scaling factors. As a result, we estimate a total of 104 976 (i.e. 2 (CH_4_ or CO_2_) × 108 (months) × 486 (sub-regions)) coefficients, by optimally fitting model concentrations with observations [28]. For further details, we refer the reader to [28].

We assume a fixed uncertainty of 40% for coefficients corresponding to *a priori* CO_2_ fluxes over each sub-region, and a larger uncertainty (60%) for the corresponding CH_4_ emissions. We also assume that *a priori* errors for the same gas are correlated with a spatial correlation length of 600 km and with a temporal correlation of one month. We assume that each single GOSAT proxy XCH4:XCO2 ratio retrieval has an uncertainty of 1.2% to account for possible model errors, including the errors in atmospheric chemistry and transport. We assume uncertainties of 0.5 ppm and 8 ppb for the NOAA *in situ* observations of CO_2_ and CH_4_, respectively. Following our previous work [28], we assume a model error of 1.5 ppm and 12 ppb for CO_2_ and CH_4_, respectively. We adopt a larger percentage value for the CH_4_ model error to account for difficulties in modelling chemical sinks of atmospheric CH_4_ [61,63].

## 3. Results

Here, we report global CH_4_ fluxes and how they vary across zonal bands, progressively ascribing values to smaller geographical regions. For the sake of brevity, we focus our attention on a few geographical regions and refer the reader to other papers dedicated to changes elsewhere (e.g. [9,10,30,32,36]). We also highlight the ability of the newer TROPOMI instrument to identify example diffuse emissions from Australian coal mining.

### (a) Global and continental net CH_4_ budgets

**Figure 2.**
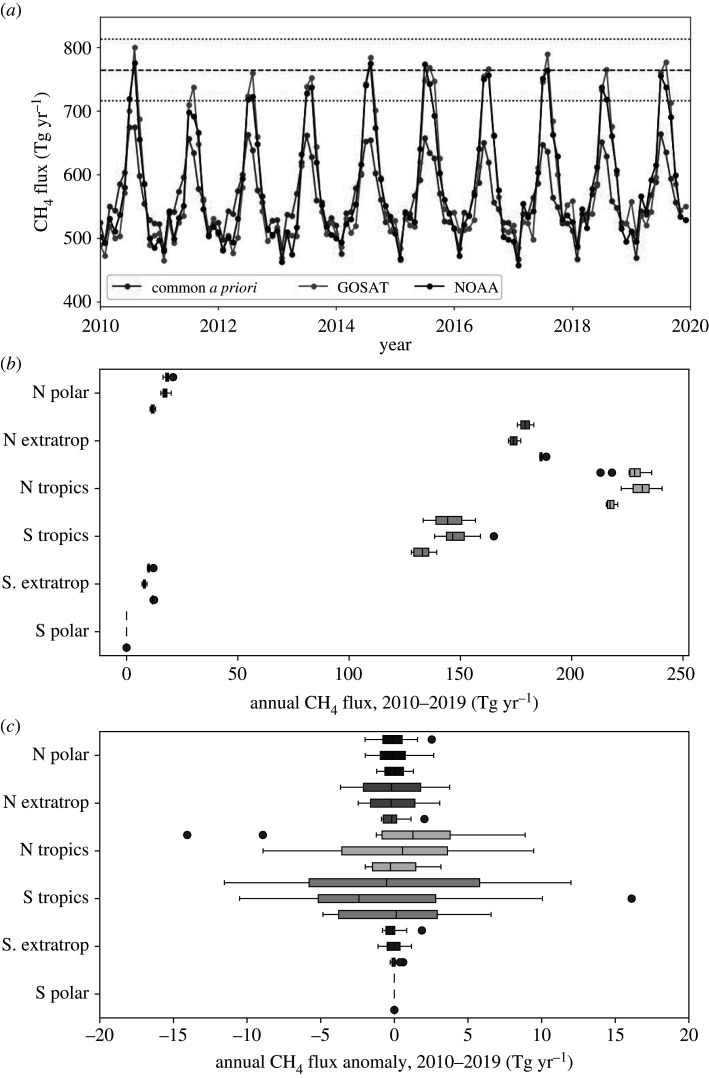
(*a*) Time series of global monthly methane fluxes (Tg yr^−1^) inferred from GOSAT and NOAA methane measurements from 2010 to 2020, and the corresponding common *a priori* values. The corresponding annual methane fluxes (Tg yr^−1^) are reported in table 2. The blue dashed and dotted horizontal denote the 2010–2019 mean seasonal peak value and the ±1-σ values, respectively. (*b*) Box and whiskers plot of the annual mean methane fluxes (Tg yr^−1^) from 2010 to 2019. The top, middle and bottom values in each triplet correspond to fluxes inferred from GOSAT and *in situ* data, and to the common *a priori* data. Estimates are described across $30^{\circ }$ zonal bands. (*c*) The corresponding annual mean anomalies, calculated by removing the 2010–2019 mean flux from all years. Red dots denote outliers that lie outside 1.5× the inter-quartile range. (Online version in colour.)

Figure 2*a* and table 2 show global emission budgets inferred using NOAA *in situ* and GOSAT CH_4_ from a common *a priori* estimates. Generally, we find that the global annual *a posteriori* estimates are within 1% of *a priori* values at the start of the decade and typically higher by 5% (≃30 Tg) after 2014. This difference increases to 10–20% after 2014 for tropical *a posteriori* estimates. Figure 2*a* shows that this annual increase mainly reflects changes during boreal summer months. There are also differences between *a priori* and *a posteriori* values during austral summer months but they are generally smaller. On this global scale, there is excellent agreement (less than 1%) between emissions inferred from *in situ* and GOSAT data, as expected, as they are determined by global mass balance.

Figure 2*b* shows the *a priori* and *a posteriori* statistics of annual CH_4_ fluxes integrated over $30^{\circ }$ zonal bins. We find agreement in the broad latitudinal distribution of CH_4_ fluxes. The largest fluxes are found in the northern tropics, northern extratropics ($30^{\circ }N\text{–}60^{\circ }N$), and the southern tropics. Emissions from the poles and southern extratropics are comparatively small. Again, the two sets of *a posteriori* estimates are statistically consistent, with increases relative to the *a priori* in the tropics and a decrease in the northern extratropics. Figure 2*c* shows annual anomalies relative to the corresponding the *a priori* and *a posteriori* 2010–2019 annual mean values. The largest anomalies are over the southern tropics ($0^{\circ }S\text{–}30^{\circ }S$) and the northern tropics ($0^{\circ }N\text{–}30^{\circ }N$) with significant variations over the northern extratropics and northern pole. The NOAA *a posteriori* fluxes show the largest relative variations over the northern tropics and GOSAT shows the largest relative variations over the southern tropics.

**Figure 3.**
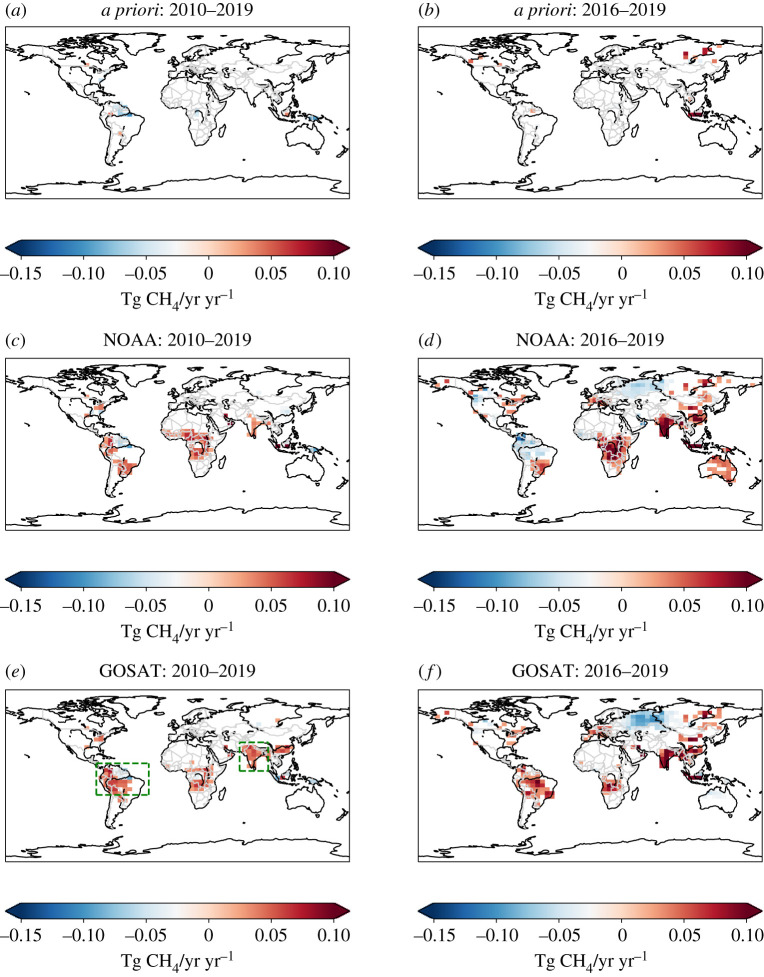
Linear trend estimates, determined by the Siegel repeated median estimator, ($\mathrm{Tg}\;{\mathrm{CH}}_{4}/{\mathrm{yr}\;\mathrm{yr}}^{-1}$) of *a posteriori* CH_4_ fluxes taken from (*a*,*b*) *a priori* inventories, and inferred from (*c*,*d*) NOAA *in situ* measurements and from (*e*,*f*) GOSAT column data for (*a*,*c*,*e*) 2010–2019 (n = 120) and (*b*,*d*,*f*) 2016–2019 (n=48). We discard absolute trends $<0.025\;\mathrm{Tg}\;{\mathrm{CH}}_{4}/{\mathrm{yr}\;\mathrm{yr}}^{-1}$ to emphasize the largest positive and negative trends. Green dashed boxes denote our definitions of tropical South America and the Indian subcontinent used in subsequent analyses. (Online version in colour.)

Figure 3 shows the Siegel linear trends for *a priori* and *a posteriori* CH_4_ fluxes during 2010–2019 and during the second half of that decade to minimize the impact of the El Niño. We use the Seigel non-parametric estimator [64] to fit a line to our data because the method is less sensitive to outliers that would otherwise compromise the linear trend estimate and the resulting estimated trend has a lower variance; we find similar trend estimates using the Theil-Sen estimator. In our 2010–2019 calculations (n=120), we want to estimate the secular trend without considering the large-scale perturbation from, for example, the 2014–2016 El Niño. By definition this approach also removes large CH_4_ pulses that we have previously attributed to anomalous precipitation [10]. We discard trends with an absolute value less than $0.025\;\mathrm{Tg}\;{\mathrm{CH}}_{4}/{\mathrm{yr}\;\mathrm{yr}}^{-1}$ to focus on the largest positive and negative trends. We find that there are small, localized *a priori* trends that are mainly associated with fire inventories that are already influenced by satellite data. By contrast, our *a posteriori* fluxes 2010–2019 (figure 3*c*,*e*) show large positive and negative trends across the tropics, particularly over tropical South America, Central Africa, India and southern China. Trends are generally larger for GOSAT, but their broad distribution is similar for both NOAA and GOSAT, which is remarkable given the comparatively small number of NOAA data over the tropics.

When we consider only the second half of the decade (2016–2019, n=48) we find that the trends over the tropics are larger and there are more extra-tropical regions with trends greater than $0.025\;\mathrm{Tg}\;{\mathrm{CH}}_{4}/{\mathrm{yr}\;\mathrm{yr}}^{-1}$ (figure 3*d*,*f*). By contrast, trends driven by the *a priori* inventories (figure 3*b*) are mostly limited to small geographical regions over North America and Siberia. We also find broad geographical agreement between *a posteriori* fluxes inferred from NOAA and GOSAT data, although there are differences in the magnitude of trends (e.g. India) and there is widespread discrepancy across tropical South America and Australia. The largest negative trend is over Russia, west of the Ob River. We now investigate in more detail the temporal variations in estimated fluxes over India and tropical South America.

### (b) Tropical South America

**Figure 4.**
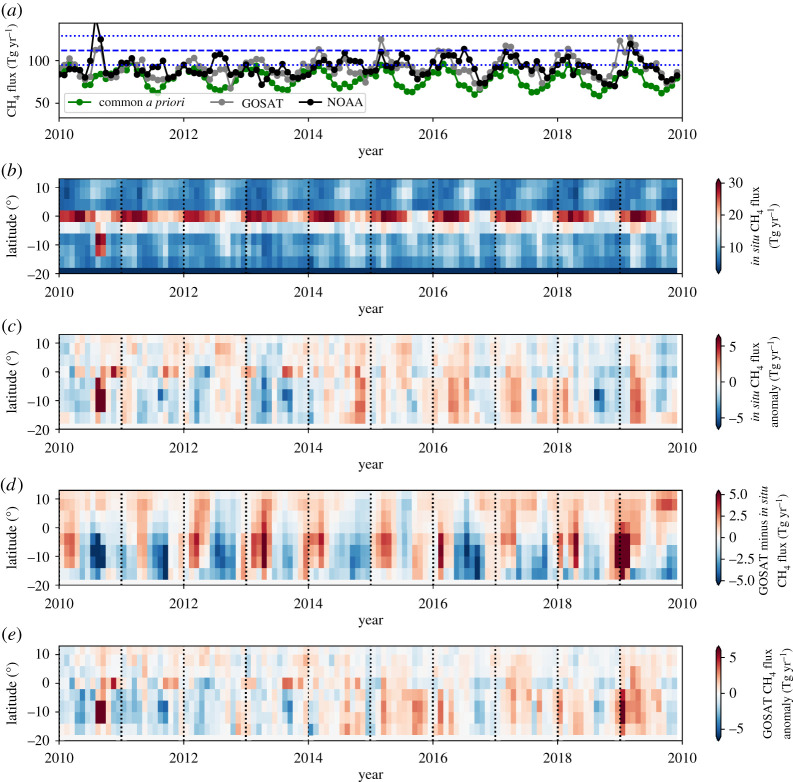
(*a*) Monthly *a priori* and *a posteriori* CH_4_ flux estimates (Tg yr^−1^) for tropical South America, (broadly defined by $30\text{–}85^{\circ }W$, $-20^{\circ }S\text{–}13^{\circ }N$) from 2010 to 2019. *A posteriori* estimates are inferred from (black) NOAA *in situ* measurements (black) and (grey) GOSAT column measurements (grey) using (green) common *a priori* estimates. Corresponding annual flux estimates are denoted by squares. (*b*) *A posteriori* flux estimates inferred from *in situ* data as a latitude-time Hövmoller plot, and (*c*) the corresponding monthly flux anomalies relative to 2010–2019 monthly means. (*d*) Monthly *a posteriori* flux estimates inferred from GOSAT data relative to the monthly *in situ a posteriori* estimates. (*e*) Monthly GOSAT *a posteriori* flux anomalies relative to 2010–2019 monthly means. (Online version in colour.)

Figure 4*a* shows the monthly and annual time series of *a priori* and *a posteriori* CH_4_ fluxes (Tg yr^−1^) over tropical South America (broadly defined by $30\text{–}85^{\circ }W$, $-20^{\circ }S\text{–}13^{\circ }N$) from 2010 to 2019; the corresponding annual values are also reported in table 2. Even on this large spatial scale there are periods of substantial deviation from fluxes inferred from NOAA and GOSAT and the common *a priori* inventory, most notably during the 2014–2016 El Niño, suggesting both these data contain information about this broad geographical region. We find a strong seasonal cycle of CH_4_ emissions, particularly at equatorial latitudes (figure 4*b*), that peaks in the first half of each calendar year and is driven by rain-fed wetland emissions. This seasonal cycle is less obvious for the regional monthly means (figure 4*a*).

**Table 2.** Annual *a priori* and *a posteriori* CH_4_ fluxes and their 1-σ uncertainties (Tg yr^−1^) for the globe, tropical South America and subcontinental India.

annual CH_4_ emissions (Tg yr^−1^)									
	global			tropical S. America			Indian subcontinent		
		*a posteriori*			*a posteriori*			*a posteriori*	
year	*a priori*	*in situ*	GOSAT	*a priori*	*in situ*	GOSAT	*a priori*	*in situ*	GOSAT
2010	571.0±20.8	572.6±10.4	568.4±6.0	81.3±6.8	91.0±5.3	87.1±3.1	47.8±4.1	50.4±2.4	50.3±1.4
2011	560.1±19.1	560.1±10.2	562.1±5.9	76.9±5.7	86.8±4.8	83.7±3.1	48.5±4.2	51.4±2.5	50.3±1.4
2012	562.6±19.2	565.2±10.0	561.0±5.9	74.6±5.7	87.6±4.8	85.4±3.1	48.4±4.2	53.0±2.5	52.1±1.5
2013	561.0±19.2	563.8±10.0	568.0±5.9	76.3±5.7	82.2±4.8	86.7±3.1	48.6±4.2	51.9±2.5	50.0±1.4
2014	564.8±19.2	590.0±9.9	585.3±5.8	75.9±5.7	91.1±4.8	88.4±3.1	48.3±4.2	52.7±2.4	53.4±1.5
2015	562.9±19.2	593.5±9.7	597.6±5.9	73.4±5.7	88.6±4.9	91.1±3.1	48.7±4.2	53.6±2.4	53.4±1.5
2016	554.5±19.2	583.0±9.9	582.4±5.8	73.6±5.7	90.4±4.8	87.1±3.1	48.9±4.2	52.0±2.4	53.2±1.5
2017	553.7±19.2	588.0±9.9	588.2±5.8	73.1±5.7	89.5±4.8	90.0±3.1	48.8±4.2	54.2±2.5	58.0±1.5
2018	553.5±19.2	582.8±9.9	586.8±6.0	72.4±5.7	83.0±4.8	87.3±3.1	48.8±4.2	54.5±2.5	57.1±1.5
2019	560.5±19.2	597.1±9.9	598.9±6.1	73.4±5.7	85.7±4.8	93.1±3.1	48.8±4.2	57.3±2.5	58.1±1.5

Broadly, below the equator, GOSAT *a posteriori* fluxes are higher than fluxes inferred from NOAA data in the first half of each calendar year, usually dominated by wetland emissions during regional wet seasons, and lower during the second half of the calendar year when emissions are dominated by dry-season fire emissions that tend to be further south. Above the equator, we find the highest emissions are during the second half of the year and focused over the Orinoco River floodplain that spans Venezuela and Colombia.

The 2010 CH_4_ pulse represents the largest anomaly in the decadal record for emissions over Tropical South America inferred from NOAA (figure 4*b*,*c*) and for GOSAT (figure 4*e*), but the distribution of these pulses are spatially distinct from each other (figure 4*d*) and from the *a priori* inventory (not shown). The spatial distribution of the CH_4_ pulse during August–September 2010 inferred from GOSAT data is focused over the Amazon forest that intersects the Brazilian states of Goiás, Tocantins and Mato Grosso, and the Bolivian portion of the Amazon forest, closely resembles the distribution of maximum climatological water deficit that has been used previously as a metric for drought intensity [65] and likely due to elevated fire emissions.

The spatial distribution of elevated *a posteriori* emissions inferred from NOAA and GOSAT data during February–April 2019 closely follow the *a priori* inventory for wetlands, focused over Ilha de Marajó in the Brazilian state of Pará; Iquitos, Peru; following the Amazon river across the Brazilian state of Amazonas; and along the northern section of the Beni River in Bolivia. We do not currently have an explanation for this pattern of elevated emissions during early 2019. We find no evidence for elevated rainfall, surface temperatures or fires. Variations in wetland emissions of CH_4_ are also driven by changes in the carbon supply that supports methanogenesis. So a plausible explanation for higher CH_4_ emissions in 2019 is that elevated fire activity from the previous dry season increased the pool of carbon available for methanogenesis (per. comm.: A. A. Bloom, JPL, May 2021), but further data are needed to improve understanding of the biogeochemical processes that control Amazonian wetland emissions of CH_4_ [66].

### (c) Indian subcontinent

**Figure 5.**
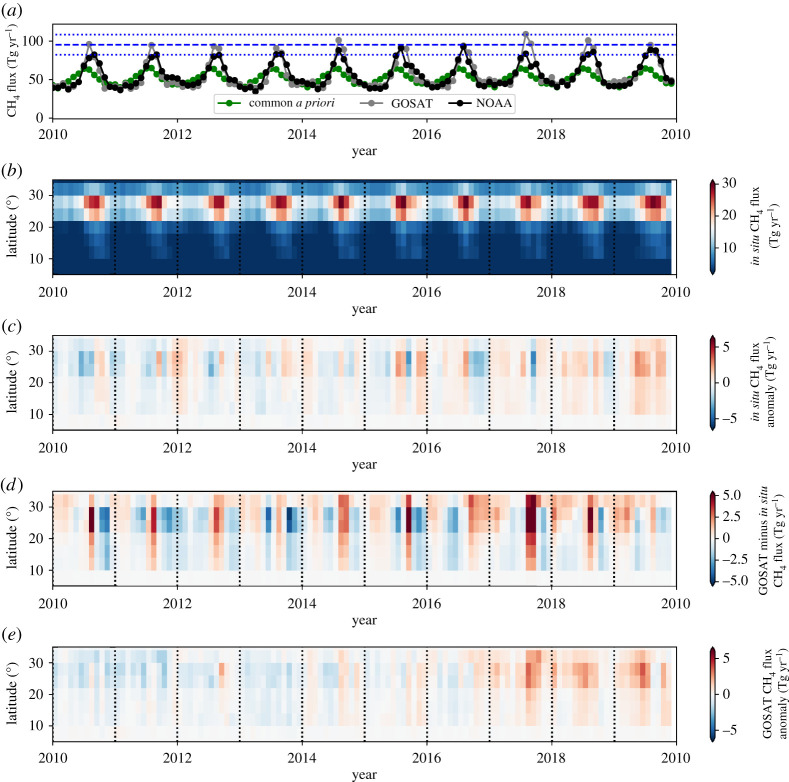
As figure 4 but for the Indian subcontinent, broadly defined as 65–95${\;}^{\circ }E$, 5–35${\;}^{\circ }N$. (Online version in colour.)

Figure 5*a* shows the monthly and annual time series of *a priori* and *a posteriori* CH_4_ fluxes (Tg yr^−1^) over the Indian subcontinent (broadly defined by $65\text{–}95^{\circ }E$, $5\text{–}35^{\circ }N$), which includes parts of Pakistan, Bangladesh, Bhutan and western Myanmar. Annual values are also reported in table 2. There is a clear regional seasonal cycle that peaks during July–October over the region broadly defined by 20–30${\;}^{\circ }$N (figure 5*b*) and 75–85${\;}^{\circ }$E (centred over Utter Pradesh) consistent with the main Kharif rice growing season that is sown in June–July and harvested in November–December. The timing of the peak is consistent with these rice plants being sufficiently mature during July–October to allow effective transmission of CH_4_, produced by rhizospheric methanogens, through their aerenchyma [67]; we acknowledge that current knowledge about plant-mediated transport of CH_4_ remains incomplete due to lack of convenient collection methods [68].

Generally, *a posteriori* estimates deviate from *a priori* estimates throughout the year with the largest values during January–October (figure 5*a*). *A posteriori* CH_4_ emission estimates inferred from GOSAT tend to be larger than *a priori* estimates during the peak of the seasonal cycle over Utter Pradesh, as described above, and comparable or slightly smaller at the seasonal trough. We find that *a posteriori* flux estimates inferred from NOAA show less year to year variability in the seasonal peaks than those inferred from GOSAT data (figure 5*a*,*d*), although their monthly anomalies with respect to their own 2010–2019 mean show some consistency during periods when the regional seasonal peaks are at their largest (figure 5*c*,*e*), e.g. during the El Niño period and 2017. In general, our year to year variations in CH_4_ fluxes up until 2015 are more consistent with those from [32] than [30]. Since 2017, GOSAT fluxes (and to a lesser extent NOAA fluxes) show a step-wise increase in emissions (figure 3) over Northeast India and northern Bangladesh, although the resolution of our *a posteriori* fluxes precludes further localization. Seasonal flooding, changes in rice production, and increased coal mining to support growing national energy demands are potential culprits but further investigation of this observation is outside the scope of this study.

### (d) New satellite data allows hotspot mapping: Australian case study

Satellite observations from the TROPOMI satellite provide daily global spatial coverage, subject to cloud cover and aerosol loading, at higher spatial resolution than previous Earth-orbiting sensors (figure 1). This high-spatial resolution allows us to focus on smaller source regions or even large individual emitters of CH_4_. There are many examples in the literature that use TROPOMI data in this way, particularly focused on the oil and gas sector that is an exemplar of a large point source of CH_4_ [69–72]. These studies have largely focused on the use of individual overpasses and plumes of CH_4_ measured by TROPOMI on certain days. However, cloud coverage can hinder regular observations of a particular source and even at the 5.5×7 km^2^ resolution of TROPOMI, the underlying source may not be resolved because the emission rate corresponds to a CH_4_ column perturbation comparable to the measurement noise in which case combining measurements collected successively over a region is required.

**Figure 6.**
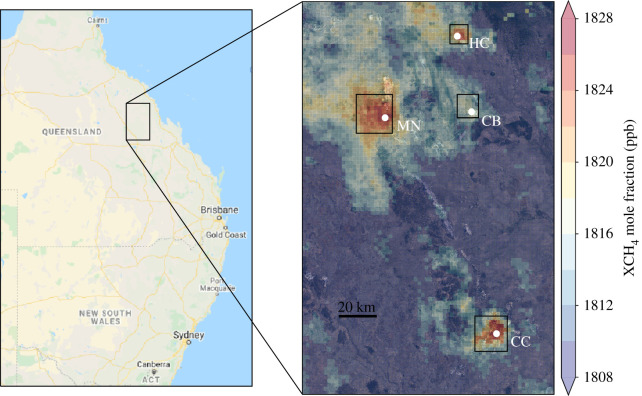
Oversampled TROPOMI column data (ppb) over the Bowen Basin in Queensland, Australia. CB, CC, HC and MN denote Coppabella, Capcoal, Hail Creek and Moranbah North/Boradmeadow (MN) mines (table 3). (Online version in colour.)

We demonstrate the capability of TROPOMI data to observe and quantify emissions on the scale of large individual coal mines. We focus on the Bowen basin region of Queensland (QLD), Australia (figure 6). Data from individual overpasses indicate the presence of significant CH_4_ sources within the Bowen basin. However, as a region containing over 40 coal mines, it is not clear from where exactly the high CH_4_ concentrations emanate. To resolve this, we use a temporal oversampling approach [73,74] to average CH_4_ column data collected from individual overpasses onto a regular fine resolution grid (in our example, $0.02^{\circ }\times 0.02^{\circ }$) to isolate the major sources of emissions within the Bowen basin. We use a simple point radius based approach to the temporal oversampling, following [74]. For each 0.02${\;}^{\circ }$ target grid cell, we find all TROPOMI pixels whose centre is within 5 km of the centre of the target grid cell. We then use one year of data to build up a $0.02^{\circ }\times 0.02^{\circ }$ grid of CH_4_ concentrations during 2019.

**Table 3.** Production statistics, reported and estimated emissions and TROPOMI annual mean enhancement from selected mines in the Bowen Basin, Queensland, Australia. Coal production statistics are taken from www.data.qld.gov.au/dataset/coal-industry-review-statistical-tables, last accessed 26th March 2021. CO_2_-equivalents are calculated using a GWP of 28 from IPCC AR5.

mine	type	annual production (Mt)	reported emissions(Mt ${\mathrm{CO}}_{2}-\mathrm{eq}$)	estimated emissions ($\mathrm{Mt}\;{\mathrm{CO}}_{2}-\mathrm{eq}$)	$\Delta X{\mathrm{CH}}_{4}(\mathrm{ppb})$
Capcoal	underground and surface	11.81	2.80	3.1±1.5	17
Moranbah North/Broadmeadow	underground	13.01	3.18	3.3±1.5	15
Coppabella	surface	3.61	0.19	0.9±0.4	7
Hail Creek	surface	7.66	0.50	1.2±0.6	12

Figure 6 shows the oversampled TROPOMI data over the northern part of the Bowen basin. Clear CH_4_ enhancements of up to 20 ppb are seen over several sets of coal mines. These mines are identified in the figure as Moranbah North / Broadmeadow (MN), Hail Creek (HC), Coppabella (CB) and Capcoal (CC). The oversampled data demonstrate the ability of TROPOMI to isolate large sources of emissions such as these mines.

To quantify the annual mean CH_4_ emissions from each of these mines, we use a simple mass balance approach, following [31]. Given an enhancement in atmospheric CH_4_ column (ΔX) over a source region, the emissions rate, Q, can be defined as

3.1 $$Q=\frac{\Delta XM_{{\mathrm{CH}}_{4}}UWp}{M_{\mathrm{atm}}g},$$

where U is the mean 10 m wind speed, W is the size of the box, p is the dry atmospheric surface pressure, g is the gravitational constant and the $M_{x}$ terms represent the molar mass of CH_4_ and the atmosphere. We use values of U and p from MERRA-2 reanalyses, as used by the GEOS-Chem model. For this illustrative calculation, we do not take into account changes in wind direction over the oversampling period.

Table 3 shows the annual mean emission estimates from each of the selected mines alongside the respective production statistics and reported annual total greenhouse gas emissions, described as CO_2_ equivalent values assuming a global warming potential of 28 [7], under the Australian reporting system for national highest emitters. We acknowledge these estimates are not directly equivalent to our CH_4_ emissions, but the majority of CO_2_-equivalent emissions from coal mines are from CH_4_ rather than CO_2_ so they can be reasonably compared.

Moranbah North and Capcoal have the largest reported emissions, reflecting that they are underground coal mines that generally emit more CH_4_ than surface mines because of the higher gas content of deeper coal seams. Our emission estimates for both these mines are broadly equivalent to the reported total. By contrast, our emission estimates for the two surface mines, Coppabella and Hail Creek, are four and two times larger than the reported amounts, respectively. This discrepancy may reflect large errors in emission factors for surface coal mines. We find that other surface mines in the region do not have similarly detectable CH_4_ enhancements, despite having larger total coal production. So our larger emission estimates may also be a result of mine-specific activities or enhanced gas content in these particular coal seams. We also acknowledge that our estimates have large uncertainties that reflect uncertainties associated with the assumed uniform wind speed, quantifying the CH_4_ column enhancement relative to the local background, and the definition of each source region. Some of these uncertainties could be reduced by using a high-resolution three-dimensional meteorological model but nevertheless the enhancements over the Bowen basin (figure 6) demonstrate the capability of the current generation of satellite data to identify the largest CH_4_ emitters so they can be compared with national reporting mechanisms (e.g. [75]).

## 4. Concluding remarks

We have shown that the added value of satellite data for understanding the contemporary CH_4_ budget is mainly from its superior spatial coverage, particularly over the tropics where there are very few *in situ* measurements. On a global scale, we find excellent agreement between CH_4_ fluxes estimated using data collected by the NOAA surface network and by the Japanese Greenhouse gases Observing SATellite (GOSAT), as expected. Differences begin to appear when these *a posteriori* fluxes are described on $30^{\circ }$ latitudinal bins but they are mostly within the associated *a posteriori* uncertainties. Even on large continental scales, long-term trends (2010–2019) in emissions from NOAA and GOSAT data are mostly consistent. It is only when we investigate shorter-term variations and sub-continental spatial scales that we see a significant discrepancy between the distribution and magnitude of CH_4_ flux estimates. We demonstrate this by examining fluxes over tropical South America and the Indian subcontinent, regions that have recently experienced large-scale climate perturbations. Recent increases in the global atmospheric CH_4_ growth rate are linked to large and rapid changes in emission sources, particularly over tropical continents where GOSAT can provide more spatially resolved information than NOAA data.

For the sake of brevity, we have limited our analysis to CH_4_ column data and consulted other data as part of the narrative. In practice, we have a wealth of *in situ* and satellite data to help attribute observed changes in CH_4_ to changes in fire, hydrology and anthropogenic emissions [76]. Integrating those auxiliary data into a coherent narrative about changing CH_4_ emissions is already possible. Formally integrating data that describe the carbon cycle and water, for example, within a Bayesian framework represents an important next step for the community. Only with this approach can we move towards a more process-level understanding of, say, wetland emissions that can then be challenged and refined with targeted fieldwork measurements. This formal approach requires that we characterize the error budget of the remotely sensed data, which requires a sustainable and transparent ground-truthing framework (e.g. [17,77,78]).

Newer instruments such as TROPOMI that have better daily coverage and finer spatial resolution open up new research directions. For example, we used these data to estimate diffuse emissions from Australian coal mines. Other groups have already started using these data to study emissions from large urban centres, power plants that effectively represent large, fixed-point sources, and to improve understanding of the controls of wetland emissions, moving beyond what can be achieved using GOSAT. The next generation of satellite instruments, e.g. GHGSat (www.ghgsat.com/), MethaneSat (www.methanesat.org/), Space Carbon Observatory (https://scarbo-h2020.eu), and the constellation of sensors aboard the Copernicus CO_2_ service, will dramatically increase the volume of high-spatial resolution quality CH_4_ data.

**Figure 7.**
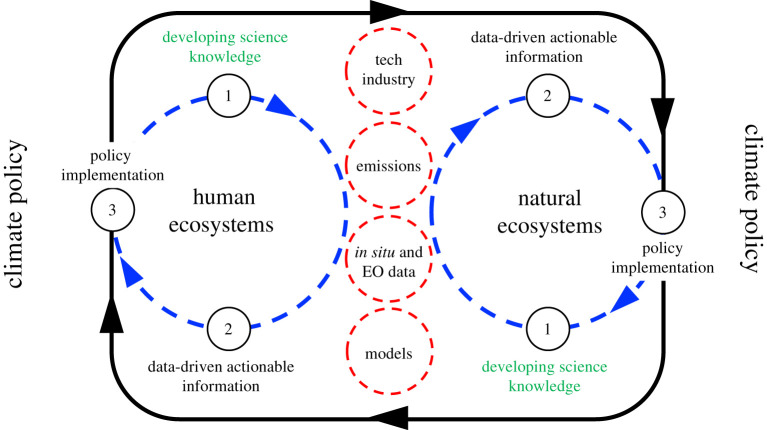
The links and exchanges that need to work to produce data-driven actionable information for the development of climate policy that simultaneously addresses human and natural ecosystem emissions of CH_4_. The numbers in each circle indicate the initial order of activities. (Online version in colour.)

The grand challenge is to use data to improve predictive Earth system models so they can better understand what is in store for us and to develop effective climate policy (figure 7). To achieve the necessary but ambitious goals of the Paris Agreement requires that we understand emissions from human ecosystems (e.g. urban centres, oil and gas industry, food production) *and* natural ecosystems (e.g. wetlands). They represent complementary measurement and analysis challenges. On spatial scales of our largest cities (less than 100 km) we need to make better use of new technology alongside more established instruments, taking advantage of fixed (e.g. buildings [79]) and moving (e.g. transport [80]) urban structures. A sustainable global observing system requires a business model. We propose that the global scale observing backbone, delivered by calibrated ground-based networks and satellites, should be funded by public money, reflecting the climate commons. Urban ecosystem measurement systems, including commercial satellites, should be funded by emitters and climate finance and by potential customers, e.g. insurance industry and hedge funds, to promote decarbonization projects. More accurate information about city emission trends will help create new markets that are not covered by current carbon trading schemes.

It is encouraging that most of the technological and scientific expertise necessary to address our challenges already exists in different disciplines and sectors (figure 7). Part of our transformational challenge will be how to harness that expertise. Meeting the demands of the Paris Agreement also requires major structural changes in the way we live, the way we produce and consume energy, and the way we do business. Collectively, these will be generation-defining changes.

## Data accessibility

All the data and materials used in this study are freely available. The NOAA ObsPack data products (https://esrl.noaa.gov/gmd/ccgg/obspack/) are available subject to their fair use policies. The University of Leicester GOSAT Proxy v9.0 XCH4 data are available from the Centre for Environmental Data Analysis data repository [39]. The presented material contains modified Copernicus TROPOMI CH_4_ data, available from ftp://ftp.sron.nl/open-access-data-2/TROPOMI/tropomi/ch4/. The GEOS-Chem model code is available at http://acmg.seas.harvard.edu/geos/.
