# Correction to ‘The added value of satellite observations of methane for understanding the contemporary methane budget’

**DOI:** 10.1098/rsta.2021.0421

**Published:** 2022-03-07

**Authors:** Paul I. Palmer, Liang Feng, Mark F. Lunt, Robert J. Parker, Hartmut Bösch, Xin Lan, Alba Lorente, Tobias Borsdorff

*Phil. Trans. Soc. A* **379**, 20210106. (Published Online 27 September 2021). (doi:10.1098/rsta.2021.0106)

In the original version of this article, figure 2*b*, figure 2*c* and the corresponding caption used a unit of 10^12^ Tg yr^−1^ to describe annual methane fluxes and annual methane flux anomalies. The correct unit should be Tg yr^−1^. This has now been corrected.

**Figure 2.**
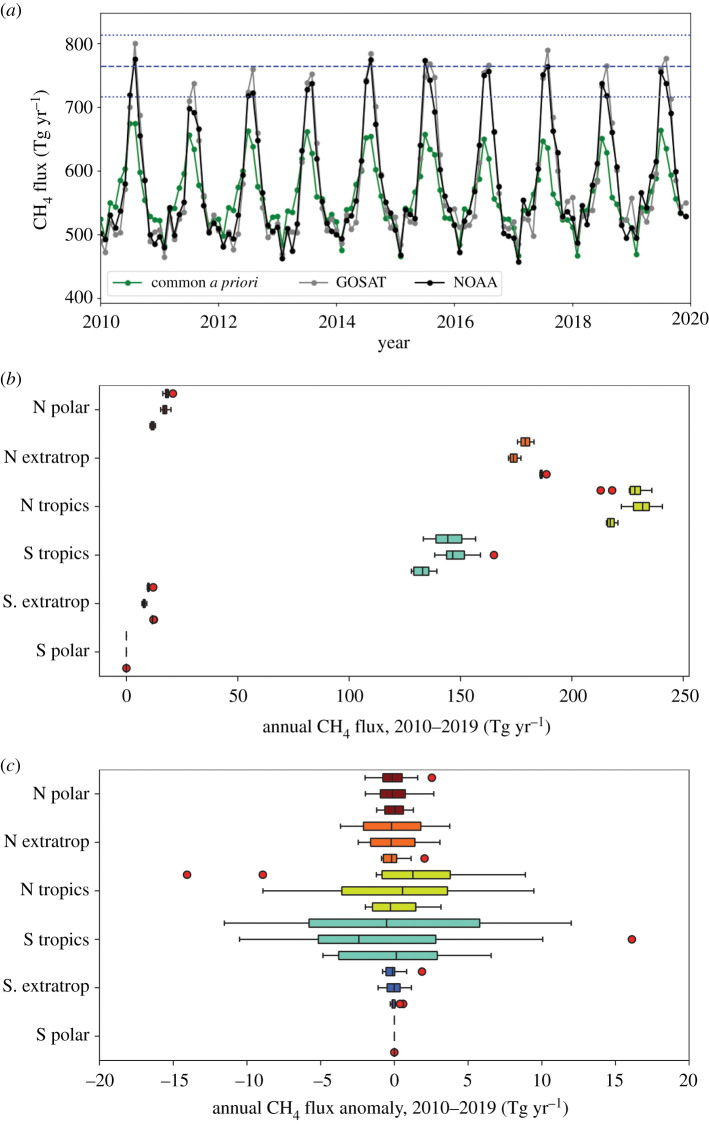
(*a*) Time series of global monthly methane fluxes (Tg yr^−1^) inferred from GOSAT and NOAA methane measurements from 2010 to 2020, and the corresponding common *a priori* values. The corresponding annual methane fluxes (Tg yr^−1^) are reported in table 2. The blue dashed and dotted horizontal denote the 2010–2019 mean seasonal peak value and the ±1 −σ values, respectively. (*b*) Box and whiskers plot of the annual mean methane fluxes (Tg yr^−1^) from 2010 to 2019. The top, middle and bottom values in each triplet correspond to fluxes inferred from GOSAT and *in situ* data, and to the common a priori data. Estimates are described across 30° zonal bands. (*c*) The corresponding annual mean anomalies, calculated by removing the 2010–2019 mean flux from all years. Red dots denote outliers that lie outside 1.5×the inter-quartile range. (Online version in colour.)

